# A proof of concept approach to quantify body schema using local Shannon entropy

**DOI:** 10.1038/s41598-026-59332-x

**Published:** 2026-06-22

**Authors:** Serena Basta, Eleonora Montagnani, Monica Gori

**Affiliations:** 1 Unit for Visually Impaired People (U-VIP), Fondazione Istituto Italiano di Tecnologia, 16163 Genoa, Italy; 2 Department of Informatics Bioengineering, Robotics and Systems Engineering, University of Genoa, 16145 Genoa, Italy; 3 Institute for Human and Machine Cognition (IHMC), 40 South Alcaniz St., Pensacola, FL 32502 USA

**Keywords:** Local Shannon entropy, Body schema, Drawing, Stroke length, Physical activity, Age, Stroke entropy, Neuroscience, Psychology, Psychology

## Abstract

Body schema is shaped by sensorimotor interactions with the environment and supports coordinated movement. Measures for quantifying body schema are limited, and little is known about how lifestyle factors influence body schema organization. We propose Shannon Entropy of digital drawing strokes as a quantitative data-driven measure of body schema complexity. Forty-four adults drew themselves (body drawing) then a flower (control drawing), and completed the International Physical Activity (PA) Questionnaire. Local Shannon Entropy was computed using a block-based, data-driven algorithm and residualized for stroke length. Body drawings showed lower entropy than control drawings, offering preliminary evidence that stroke entropy may capture body-specific differences in representational complexity, meriting further investigation with counterbalanced designs. Residual entropy decreased with age, suggesting stabilization of body schema in older adults. PA was evaluated via standard Physical Activity Levels (PAL) and data-driven tertiles. PALs showed no significant effects, likely due to sample disproportion. Tertile analysis revealed a significant interaction between PA and age: more physically active participants maintained stable drawing entropy with age, suggesting PA may be associated with age-related reductions in body representation. Findings suggest stroke entropy as an interesting method for future validation studies examining the role of PA in maintaining body representations across adulthood.

## Introduction

Body representation is defined as the representation of an individual’s entire body, referring not only to the size or shape but also to all the necessary information for interacting with the environment^1^. Essentially, body representation is divided into *body image*, concerning the perceptual identification and recognition of the body^2^, and *body schema*, which is defined as the representation of a body in action^3^. The body schema is closely related to the sensorimotor system in the brain and it is continuously shaped through interactions with the environment^4,5^.

Over time, as individuals grow, the body schema gradually stabilizes, and this process likely continues until around 9–10 years of age^6^. It is assumed that this consolidation process occurs through regular movement and interaction with the environment, reinforcing the individual’s body representation^7^.

The stability of the body schema is influenced by brain plasticity and sensory modalities, which contribute to motor control and adaptation^6^. While the general trajectory of body schema development is known, it is still uncertain how it is influenced by various factors such as age, strength, or physical activity. Previous works have found that body schema is indeed influenced by the movement because it is generated by the motor system, and it is, therefore, an integral part of it^3^, as shown by tests for its evaluation^8–11^. It is known that regular and long-term physical activity (PA) enhances body perception and awareness^12,13^. This is in agreement with the description of Sirard and Pate, who considered PA as any type of body motion that causes an expenditure of energy^14,15^. However, other factors can also influence the representation of the body across the lifespan, such as demographic characteristics. For example, sex differences have been shown to affect girls’ body schema differently from boys^16–18^. On the other hand, age is also an important factor to consider in body schema research^19^. Although body schema begins to form during development, evidence suggests that body representations may also change later in life, with age-related modifications in sensorimotor processing and body representation abilities observed in adults^20^.

There are many test available to explore body schema features, including motor imagery tasks^8^, mental rotation^9^, mental production^11^, and haptic stimulation^10^. The Drawing test is a simple and quick tool that can be used for assessing not only body schema but also psychological processes and cognitive abilities. Over the years, many types of drawing tests have been developed. *Human Figure Drawings test* (HFDs) was the earliest scoring system used to assess children’s drawings since 1962^21–23^, standardized by Goodenough with the *Draw-a-man test* (DAMT)^24^ used as a measure of intelligence. The test was later revised and updated by Harris^24^ resulting in the *Goodenough-Harris-Drawing Test* (GHDT), but it lacked a comprehensive scoring system until the developed *Draw a Person: A Quantitative Scoring System* (DAP: QSS)^25^. The evolution of drawing tests ended with the development of the *Draw a Person: Intellectual Ability Test for Children*,* Adolescents and Adults* (DAP: IQ)^8^, including an age range from 4 to 90 years, complementing previous knowledge and methodologies. Human figure drawing is widely used in clinical settings for self-expression^26,27^. The limitation of the drawing test is related to the difficulty in its quantification. For example, Nuara and colleagues^27^ used an asymmetry index to measure the difference in upper limb length of children with hemiparesis, using a manual measurement based on the differences between limb dimensions, expressed as a percentage of their average. Such an approach is not only time-consuming but also highly operator-dependent, making it prone to errors. As an alternative, Martinelli et al.^26^ developed a program to automatically extract dimensions of body segments, making the process faster and more accurate than measuring body feature dimensions by hand. However, literature has never explored the potential use of the entropy of digital drawings in the field of body schema, which could yield meaningful results relative to self-representation.

Specifically, Shannon Entropy measures the complexity and uncertainty of the amount of information contained in a string, where each symbol has a probability of occurring. Shannon Entropy has been applied to various fields, including brain evaluation for surgery, disease progression prediction, and image analysis for quality assessment, segmentation, and cryptography^28–30^. This application is based on the probability distribution of pixel values. In information theory, a higher level of entropy indicates randomness in the system, while a lower level of entropy describes a more predictable and stable state^31^. Assuming this, entropy could reflect information about the organization of the body schema, with low and high entropy values of the drawing strokes being potentially associated with a more predictable or less structured and organized representation of the self, respectively. However, Shannon Entropy of drawing strokes has never been estimated from digital drawings to study the body schema. Therefore, we developed and adopted a data-driven algorithm, which introduces a local entropy-based framework building on previous works using *non-overlapping* block sizes^30,32,33^. With this, the aims of our study were twofold: (1) determining whether using the Local Shannon Entropy of drawing strokes could be considered as a potential and preliminary measure of body schema, and (2) investigating the impact of various predictors related to PA and demographics upon the entropy of drawings, which is a tool notoriously used to assess the body schema.

## Methods

### Participants

Participants were recruited at the Italian Institute of Technology in Genoa between April and December 2024, and they were naïve to the experimental procedures. All participants were adults with no history of neurological, sensory disease, or acute pain or musculoskeletal injury. Individuals with expertise in drawing were excluded. A total of 44 volunteers were recruited and included in the analyses of this work (22 F, mean age: 38.07, SD 12.7; 22 M, mean age: 35.39, SD 10.4 y. o). The study was approved by the Territorial Ethics Committee of the Liguria Region (2/2020—DB id 10213) on 18 December 2023, and written informed consent was obtained from all participants in accordance with the Declaration of Helsinki.

### Apparatuses

We used an Apple iPad (LED 10.2′′,17.41 × 25.06 cm) and an Apple Pencil for the drawing task. The stroke width of each drawing was fixed at 1 pixel to avoid its influence on further calculations. We used a blank page in the Notability application (version 11.0), developed by Ginger Labs^34^, with display dimensions of 1700 × 2232 pixels and a bit depth of 8.

To measure grip strength, we adopted a Handgrip Dynamometer MAP 80K1 by Kern^35^, with a maximum weight capacity of 80 kg. This tool has been shown to have a significant correlation with physical state^36,37^ and therefore it is considered a reliable measure of this parameter. To test the grip strength of the dominant limb, handedness laterality was also assessed using the Edinburgh Handedness Inventory^38^. To quantify PA, a short form of the International Physical Activity Questionnaire (IPAQ) was used^39^, consisting of seven self-reported item regarding time spent performing PA over a week. In particular, our analyses focused on two items from the IPAQ: (1) the Physical Activity Levels (PAL) expressed as low, medium and high, and categorized based on multi-item calculation within the IPAQ; (2) Metabolic Equivalent of Task (MET), a physiological continuous unit that expresses the energy cost of PAs relative to resting metabolic rate, here calculated based on the minutes of activity performed per week^40^.

### Procedure

Each participant was asked to sit on a chair placed in front of a table. Using the iPad, participants were first asked to draw themselves within five minutes. To standardize the drawings, participants were asked to depict themselves wearing only a T-shirt and shorts. After drawing themselves, participants were also asked to draw a flower as a control stimulus, to understand whether the entropy parameter would differ between drawings of a self-portrait and a common icon. When participants finished drawing, grip strength assessment was conducted in a seated position for both the dominant and non-dominant hands, with shoulders adducted, neutrally rotated, and arms parallel to the floor. Three measurements were taken at 20-second intervals between them, and the highest value of the three was recorded^41^. The handedness laterality test was performed to establish the dominant and non-dominant hands. Only the measurements from the dominant hand were considered for analysis. Finally, the IPAQ test was administered.

### Algorithm implementation and data pre-processing

To analyze the entropy of drawing strokes from both body and control conditions, we developed a data-driven algorithm in MATLAB 2023b (The MathWorks, Natick, USA) designed to compute Local Shannon Entropy of pixel images, based on the work of Wu, Zhou^30^. This approach employs a non-overlapping block size to evaluate the entropy of drawing strokes.

**Fig. 1:**
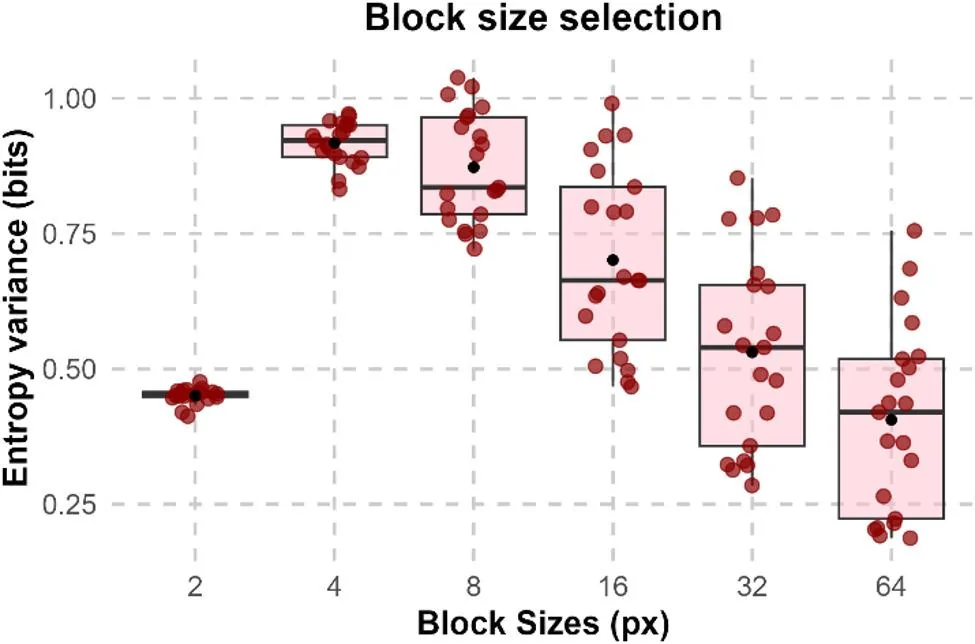
Example of distribution of entropy variance (bits) calculated across the digital drawing of flower (control stimulus) for the different candidate block sizes (neighborhood of pixels) selected. The block size that captures the largest entropy variance is considered optimal as it allows to determine larger complexity features of the data set. In the case of our dataset for the control stimulus, this is represented by the 8 × 8 block size. The black dot represents the mean value calculated across all drawings.

In brief, the algorithm converts images into greyscale and evaluates entropy across a predefined set of candidate block sizes^30^, (2 × 2, 4 × 4, 8 × 8, 16 × 16, 32 × 32, and 64 × 64 pixels). Rather than imposing a fixed spatial scale a priori, the algorithm adopts a data-driven selection procedure to identify the block size that best captures the structural variability of the dataset. Specifically, the optimal block size is determined based on the amount of maximum entropy variance retained across drawings. This approach allows the entropy metric to adapt to the spatial characteristics of the images under analysis, meaning that different datasets or stimulus types may yield different optimal block sizes. In other words, we select the block size that best represents differences in image details, thereby maximizing the useful information for analysis. An example application to flower drawings is shown in Fig. 1.

Once this passage was complete and the optimal block size identified (in the case of the control stimulus, the optimal block size resolution was 8 × 8), we used it to compute Local Shannon Entropy within each overlapping block size by rearranging local pixels of the matrix into 1D arrays. More specifically, the computation of entropy would assign the same value to each pixel within a block, and then the mean entropy is calculated among all blocks in the drawing. Because there were blocks containing only zero values (undrawn areas or partial edges of the blocks), these were excluded from the average entropy calculation. Below, we report examples of the visual resolution of the calculation of Local Shannon Entropy across the matricial system of the flower drawings, and we visualize the heatmaps of the entropy values calculated with all block sizes.

**Fig. 2:**
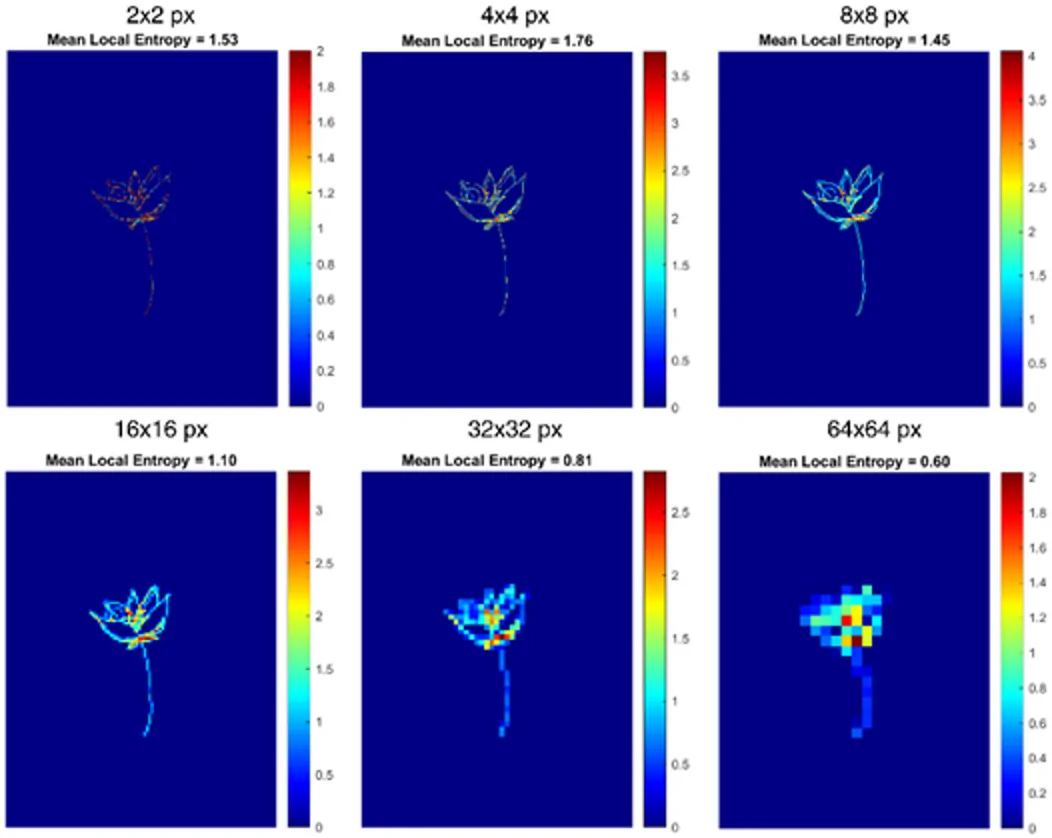
Example of the visual resolution of the flowers originating from the calculation of local Shannon entropy estimated with different block sizes. Colorbar indicate the local entropy expressed in bits.

From Fig. 2, it is important to note that block sizes of 8 × 8 pixels captured entropy values that balanced sensitivity and stability in measurement. Excessively small blocks (e.g., 2 × 2 pixels) tended to be highly sensitive to minor variations, leading to inflated entropy values that might misrepresent the underlying structural complexity. Conversely, excessively large blocks (e.g., 32 × 32 or 64 × 64 pixels) averaged over large portions of the image, potentially obscuring important localized variations. This was visible in Fig. 2, where a block size of 2 × 2 pixels would lead to losing important information of the drawing strokes. In fact, the stem of the flower was almost invisible within the heatmap. Similarly, the block size of 64 × 64 lost important details of the flower, generalizing the features among neighboring blocks of pixels.

### Data post-processing

In drawing data, entropy might not solely reflect the “stability” or “richness” of representational content; it is also mechanically affected by how much material is present in the image, particularly the total length of drawn strokes. For example, drawings that are larger, denser, or more heavily shaded naturally contain more pixels belonging to the foreground, which inflates entropy values even if the underlying representational structure is not more complex. To clarify how raw entropy values are influenced by basic structural properties of the drawings, we quantified the amount of drawn material present in each image by computing *stroke length*, operationalized as the total number of foreground pixels in the binarized drawing. Stroke length provides an objective measure of drawing quantity and is essential for distinguishing between entropies arising from representational complexity versus entropy driven simply by the amount of ink on the page. For each PNG image, we first converted the original RGB drawing to grayscale to remove color information while preserving intensity structure. We then applied adaptive thresholding to create a binary image in which foreground (inked) pixels were separated from the background. Adaptive binarization was chosen because it adjusts the threshold locally across the image. Once the binary image was obtained, stroke length was computed as the number of foreground pixels, which corresponds to the total pixel coverage of the drawn stroke.

### Data analysis

All statistical analyses were performed in R v4.3.2^42^. The significance threshold was set at *p* < .05. For the statistical analysis and inferential tests, the following packages were used: *stats*, *tidyr*^43^, *DAAG*^44^, *FSA*^45^, MASS, *Hmisc*^46^, *lme4*^47^, *mgcv*^48^, *visreg*^49^ and *emmeans*. For data visualization, *lattice*^50^,*ggplot2*^43^ and *ggdist*^51^.

### Entropy as a potential proxy to investigate body schema organization

Before examining higher-order predictors of drawing complexity, we conducted preliminary analyses to clarify how raw entropy values are influenced by stroke length operationalized as a basic structural property of the drawings. To quantitatively evaluate this dependency, we fit a linear mixed-effects model with entropy as the dependent variable, drawing condition (body vs. control) as a fixed effect, and stroke length as a covariate, including a random intercept for participant ID to account for within-subject dependency. This model allows us to evaluate whether condition differences exist and hold after statistically controlling for the amount of drawing, and to quantify the degree to which stroke length predicts raw entropy. We hypothesize that the control and body drawings would produce different entropy patterns, supporting the idea that Shannon Entropy of self-representation drawings can be used as an indicator to investigate body schema organization.

### The effect of physical activity and demographic variables upon the entropy of drawings

To further analyze body schema organization, it was crucial to identify whether selected variables could predict changes in the entropy of drawing strokes. To this end, a correlation matrix was conducted to identify potential predictors of entropy changes to be used within further models. A Spearman correlation analysis was carried out to explore the relationship between PA and demographic variables, chosen for the suitability in non-normally distribution. The correlation included sex, grip strength, MET, PALs, and age. Then, we selected the relevant variables to perform further analyses. To account for variability in the amount of drawn material across drawings, all analyses used an entropy measure residualized for stroke length. Controlling for this low-level structural property is essential for isolating features reflecting representational complexity rather than drawing quantity. Because the relationship between entropy and stroke length was nonlinear, we modeled expected entropy values as a smooth function of total stroke length using a Generalized Additive Model (GAM). fitted with a standard penalized regression spline (k = 5). The residuals from this model (i.e., observed minus predicted entropy) were extracted for each participant: these values represent the component of entropy not attributable to stroke length effects and therefore index structural complexity independent of drawing quantity. All subsequent analyses used these GAM-residualized entropy scores. Next, we examined whether age and PA predicted residual entropy and whether PA moderated the age–entropy association. PA was indexed using two complementary approaches:

1. *Physical activity levels (PALs) (Low*,* Moderate*,* High)*: Participants were classified using internationally recognized thresholds based multi-item calculation as defined within the IPAQ. This provides a theory‑driven, clinically interpretable PA continuum.
2. Data‑Driven Tertiles Groups: As a robustness analysis, MET‑minutes were also split into three tertiles: participants in the lowest third of MET-minutes were assigned to T1 (low activity), those in the middle third to T2 (moderate activity), and those in the highest third to T3 (high activity). These provide a balanced design, increase statistical sensitivity for interaction effects, and allow us to confirm that results do not depend on a particular PA coding scheme.

## Results

Following computation of our method, we found that the optimal block size needed for drawing of body and control drawings was 16 × 16 and 8 × 8 pixels, respectively, indicating that these were the block sizes that retained the most variance specifically for these stimuli. The demographic data, the Local Shannon Entropy values of stimuli, and the results of the grip strength and MET values test are shown in Table 1, reported as minimum and maximum values, means and standard deviations.

**Table 1:** Overview of participant demographics and variables.

Parameters	Min	Max	Mean	SD
Age (years)	21.3	67.3	36.7	11.5
MET (min/week)	0	4158	966.2	2023
Entropy body (16 × 16 px, bits)	0.9	2.9	1.4	0.42
Entropy control (8 × 8 px, bits)	1.2	2.8	1.5	0.32
Grip strength (N)	15.8	53.9	32.8	11.3
Stroke length body (n)	10,794	409,322	49,971	68022.6
Stroke length control (n)	6754	273,009	38,075	45740.1

MET, metabolic equivalent of task; px, pixel.

### Entropy as a method to evaluate body schema

**Fig. 3:**
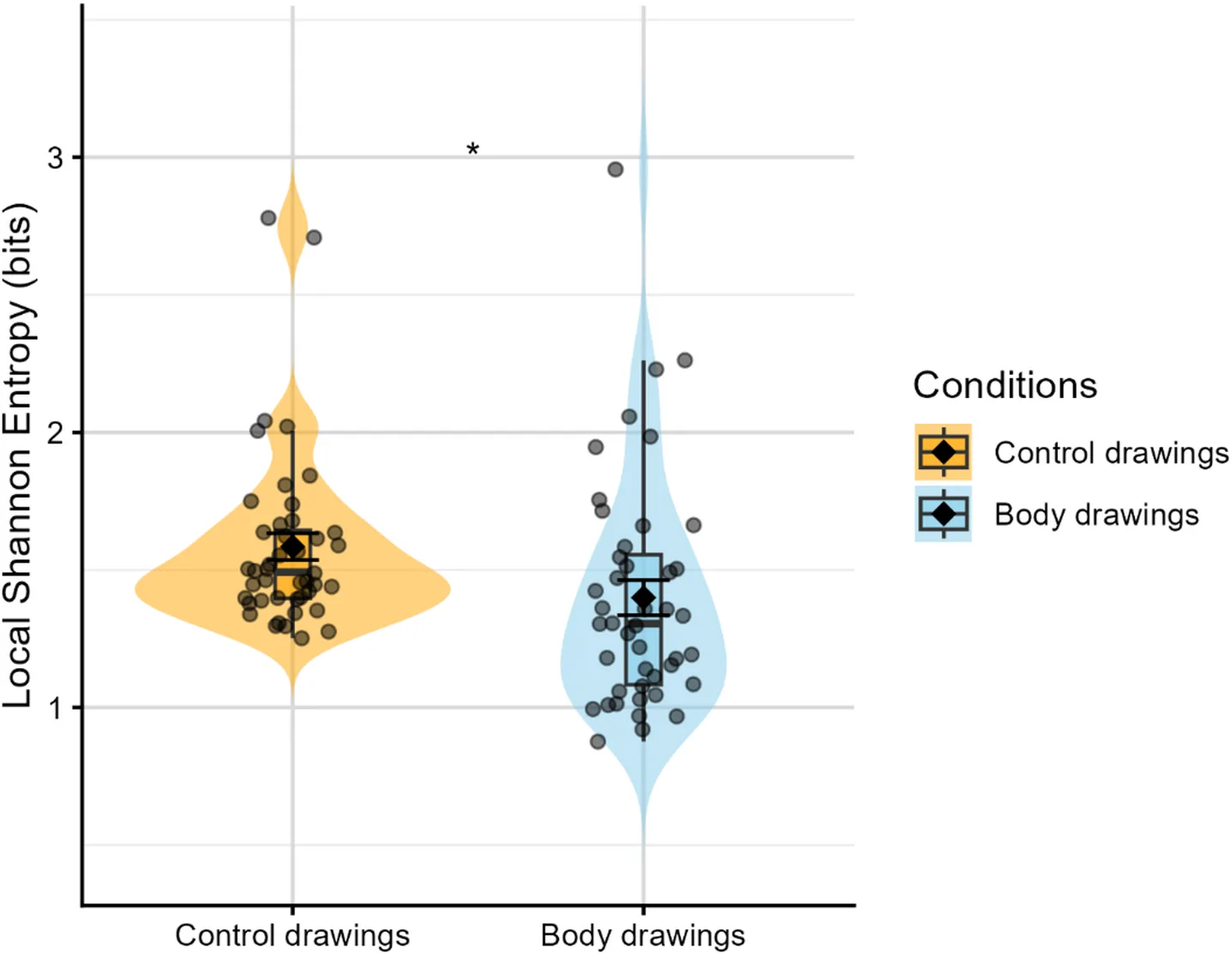
Differences in strokes entropy value between drawings. The image shows differences in the range of entropy between body and control drawings. The black dot represents the mean value, and the black bars represent the error bars.

To evaluate the possibility of using our method as a potential new metric to measure body schema, we decided to model data of this section using Local Shannon Entropy originating from a common block size of 8 × 8 pixels for both control and experimental stimuli, which guarantee methodological and statistical coherence. We observed a robust positive effect of stroke length on drawing entropy, with longer (i.e., more extensive) drawings exhibiting higher entropy values (β = 3.99 × 10⁻⁶, SE = 4.78 × 10⁻⁷, 95% CI [3.04 × 10⁻⁶, 4.93 × 10⁻⁶], t = 8.35). This indicates that entropy is strongly influenced by the physical extent of the drawing, underscoring the importance of controlling for stroke length in subsequent analyses. Critically, after adjusting for stroke length, body drawings showed significantly higher entropy than control drawings (β = 0.143, SE = 0.0315, 95% CI [0.081, 0.205], t = 4.54; Fig. 3), suggesting the presence of potential systematic differences in structural content of the two types of drawing, beyond stroke quantity. The model further included a random intercept for participant ID (variance = 0.0319), indicating meaningful between-subject variability in baseline entropy levels.

### Correlation matrix among all variables

Since MET and PAL were highly correlated (*r* = 0.90), we decided to maintain PAL as a categorical standard variable to enhance result interpretation. Similarly, strength and sex were strongly correlated (*r* = 0.84), suggesting that it would be better to maintain only one variable in the model. Grip strength was preferred because it is a more objective and direct measure than sex, and should be further examined with all features considered, as previously outlined^17,18^. Spearman correlation coefficients for sex, entropy value, strength, MET, and PAL are summarized in Table 2.

**Table 2:** Spearman correlation matrix.

Variables	Sex	Strength	MET	PAL	Age
Sex	1.00	0.82	− 0.02	− 0.16	− 0.06
Strength (N)	0.82	1.00	0.03	− 0.05	0.00
MET (min/week)	− 0.02	0.03	1.00	0.89	− 0.05
PAL	− 0.61	− 0.05	0.89	1.00	− 0.01
Age (y)	− 0.06	0.00	− 0.05	− 0.01	1.00

MET, metabolic equivalent of task; PAL, physical activity levels.

### The effect of physical activity and demographics variables on stroke entropy

Using the optimal block size of 16 × 16 pixels, we then examined whether the linear association between age and residual entropy varied across PALs. After controlling for stroke length, older participants tended to produce drawings with lower residual entropy. PAL groups trended toward lower baseline entropy at higher activity levels, but the age–entropy slope did not differ significantly across PALs. Specifically, the linear model showed a significant negative main effect of age, indicating lower residual entropy at older ages (Age: β = − 0.0156, SE = 0.006, 95% CI [− 0.027, − 0.003], t = − 2.586, *p* = 0.0103). Relative to Low activity, Moderate and High groups showed numerically lower intercepts (Moderate: β = −0.5366, SE = 0.273, 95% CI [− 1.08, 0.014], t = − 1.973, *p* = 0.056; High: β = −0.7497, SE = 0.4131, 95% CI [− 1.586, 0.087], t = − 1.815, *p* = 0.0706), although these did not meet the conventional α = 0.05 threshold. The Age×PAL interactions were not significant (Age×Moderate: β = 0.011, SE = 0.0066, 95% CI [− 0.002, 0.025], t = 1.675, *p* = 0.1042; Age×High: β = 0.0112, SE = 0.0074, 95% CI [− 0.005, 0.039], t = 1.509, *p* = 0.1397), suggesting that the negative age slope was broadly similar across PALs. Overall fit was modest (Residual SE = 0.225; R^2^=0.217, adjusted R^2^=0.111; F(5,37) = 2.05, *p* = 0.094), consistent with a reliable age effect and trend‑level differences by PAL group but no definitive moderation by PAL.

**Fig. 4:**
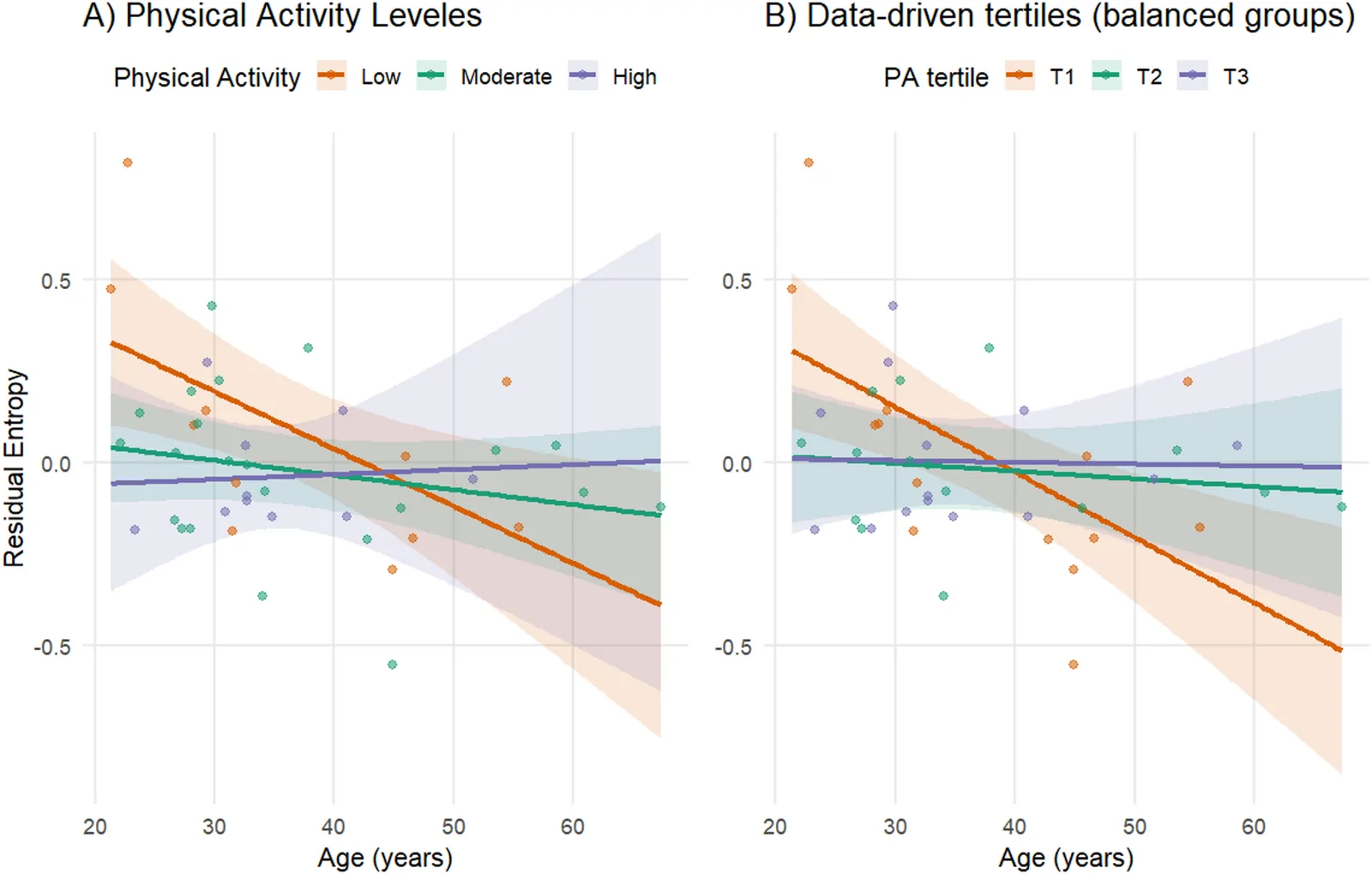
Age × physical activity effects on residual entropy: (**A**) Model‑predicted residual entropy as a function of age across PALs (Low, Moderate, High). Shaded bands represent 95% CI, and semi‑transparent points show individual data. Across PALs, residual entropy tends to decrease with age, with higher activity levels showing slightly attenuated age slopes. (**B**) The same model estimated using data‑driven tertiles of weekly MET‑minutes (T1–T3), which provide balanced group sizes. Compared with the PALs, tertiles reveal a clearer pattern: higher activity tertiles show a shallower age‑related decline in residual entropy, consistent with a moderation effect whereby greater physical activity is associated with age‑related reductions in drawing complexity after controlling stroke length.

Second, as a complementary, balanced grouping strategy, we also recoded weekly MET-minutes into tertiles (T1-T3) and re-estimated the interaction model. One observation was excluded due to missingness; the effective sample size was *n* = 43 (residual df = 39). The model was statistically significant overall, F(3,39) = 3.192, *p* = 0.0340, with R^2^=0.197, adjusted R^2^= 0.135, residual SE = 0.222). There was a significant negative main effect of age (β = −0.0238, SE = 0.00843, 95% CI [− 0.040, − 0.006], t = − 2.821, *p* = 0.00748), indicating that, on average, older age was associated with lower residual entropy (i.e., less complexity independent of drawing quantity). There was also a main effect of PA tertiles (β = − 0.3385, SE = 0.1538, 95% CI [− 0.649 − 0.027], t = − 2.201, *p* = 0.0337), reflecting systematic differences in baseline residual entropy across activity levels under the model’s linear‑ordinal coding. Crucially, the Age×PA tertiles interaction was also significant (β = 0.00892, SE = 0.00407, 95% CI [0.000, 0.017], t = 2.191, *p* = 0.0345), indicating that the age slope varies across PA tertiles, consistent with a moderation effect. Simple‑slope estimates derived from the interaction clarify this pattern. The age effect within each tertile is: T1 (low PA): β *age*(T1) = − 0.0238 + 0.00892 × 1 ≈ − 0.0149 per year ( ≈ − 0.149 per 10 years); T2 (moderate PA): β *age* (T2) ≈ − 0.00593 per year ( ≈ − 0.059 per 10 years); T3 (high PA): β *age*(T3) ≈ + 0.00299 year ( ≈ + 0.030 per 10 years). Thus, higher PA was associated with an age‑related decline in residual entropy, which could potentially indicate preserved body schema organization progressing from a clearly negative slope in the lowest tertile to a near‑flat (slightly positive) slope in the highest tertile. Overview of the section results are reported in Fig. 4.

## Discussion

In this study, we aimed to investigate whether the entropy of drawing strokes could be used as a novel informative measure of body schema, and to measure the impact of lifestyle factors such as PA and demographic characteristics upon body schema organization of healthy adults. This approach was adopted by implementing a data-driven algorithm based on previous work^30,32,33^, extending its implementation by using entropy as a quantitative index when studying body schema.

Previous research has demonstrated that the stability of the body schema is not uniform across individuals, and is shaped by different predictors, as well as by other individual differences such as sensory processing, cognition, and motor learning experiences^6,7,52^. However, the role of these predictors is still complex and not fully understood.

Concerning our first hypothesis, we found that the body and control drawings yielded significantly different entropy values after controlling for physical drawing properties (“richness” of drawn material), suggesting that body drawings may reflect body-related entropy rather than a general drawing approach. Therefore, the calculation of entropy from digital self-drawings has the potential to assess complexity features of the body schema. This could be considered an interesting advancement in the field, as it is known that quantification of drawing tests to investigate body schema is limited and challenging. In fact, previous research has used a manual assessment method^27^ or considered only isolated features of the body^26^, while our approach allows for the measurement of the complexity of the body represented in a digital drawing. Although the present findings are encouraging, it is important to note that the proposed approach was not directly validated against established measures commonly used to assess body schema.^8,9,53–56^.

Furthermore, it is important to notice that, in this study, drawings were performed in fixed order, limiting the strength of our interpretation. As such, future research is therefore required to randomize drawing of the stimuli and perform comprehensive validation against standard approaches before this measure can be more broadly adopted. Then, it is important to clarify the hypothesized connection between drawing strokes and the body, and how these strokes might be interpreted as an indirect measure of the organization of the body schema. While the stroke’s drawing may provide information about their organization and variability, it should be considered a quantitative, indirect indicator of body schema stability, as it reflects how the body is internally organized when translated into a drawing. It is also important to consider that the drawing process involves fine motor execution and visuomotor coordination, and for this reason, entropy could in principle be influenced by general motor output variability, drawing strategy, or stylistic aspects of drawing behavior. However, in the present study, the sample consisted of healthy adults without artistic training. In this population, basic drawing motor output is stable and efficient but still shows a degree of inter-individual variability, which reflects the intrinsic variability of the visuomotor control processes underlying simple line production^57^. Importantly, previous findings highlight that stroke length is a major confounding factor when interpreting raw entropy and that condition differences cannot be meaningfully evaluated without correcting for drawing quantity^58^. These considerations suggest that the observed entropy differences in this study are more likely related to representational aspects of the drawing, potentially reflecting how the body is internally structured and organized, rather than explanation about motor execution variability.

Concerning our second hypothesis, findings revealed that as individuals grow older, they tend to represent themselves less randomly. This indicates that aging may be associated with a less variable body schema, potentially as the individual becomes more familiar with their body’s capabilities and limitations. A more stable body schema could reflect a reduction in the variability of movement patterns, probably corresponding to a more refined and coordinated set of motor skills, which however can degrade during the aging process such as in elderly populations. To fully understand the implications of age-related changes in body schema, future research should explore how this impacts different aging groups, and also during childhood and adolescence, to measure body schema organization using our entropy approach, across aging and developmental stages.

Another important feature explored in this study concerns how PA influences the body schema organization. PA is known to enhance self-awareness, sensorimotor coordination, and gross motor skills^7,19^. In our study, we assessed the impact of PA using two different metrics. First, we considered the three standard PALs (Low, Medium, High) derived from the IPAQ categorization. However, this classification did not reveal significant effects or interactions with age. This lack of significance may be explained by the distribution of our sample across the PAL standard categories, which was relatively uneven and limited in size, potentially reducing the statistical power to detect meaningful differences. To address this issue, we implemented a second, data-driven approach based on the distribution of PA scores within our sample. Specifically, we divided participants into three equally sized groups using a tertile-based method. This approach allowed for a more balanced representation of participants across activity levels. Using this classification, we observed a significant interaction between PA and age: individuals with higher levels of PA (T2–T3) showed increased entropy in their drawings even with advancing age. This finding suggests that higher engagement in PA may be associated with age-related differences in the way individuals represent their bodies. In other words, maintaining higher levels of PA could contribute to preserving a more flexible and functionally organized body schema over time, potentially supporting more efficient sensorimotor representations despite the effects of aging. It should be noted that, in the present study, PA was assessed as amateur activity rather than agonistic training. Therefore, the PA levels captured here likely reflect habitual lifestyle engagement. With these findings, it would be interesting for future research to investigate body schema organization in competitive agonistic athletes, who undergo more intensive and prolonged sensorimotor training and may therefore show different patterns of body schema organization.

Furthermore, it is important to highlight that additional factors not measured in this work also play an essential role in shaping the body schema. Pain, for example^59^, has been shown to influence the mental representation of movement. This highlights the importance of considering clinical predictors, but also injury or other neurological conditions. Understanding these factors in clinical populations could provide important information into how body schema differs between individuals with neurological conditions and healthy individuals. Further research in this field could show how clinical interventions, such as physiotherapy or rehabilitation programs, could be adapted to promote a more adaptive and stable body schema.

### Limitations

While findings from this work are encouraging and demonstrate potential novelty in this field, there are some limitations that should be highlighted, which can be addressed in future research. First, the fixed drawing order (body first, then flower) represents a potential confound for the body-vs-control comparison, as practice or fatigue effects cannot be ruled out. This limits the strength of conclusions drawn from this comparison, and future studies should randomize drawing order to address this issue. Then, collecting additional information as history of musculoskeletal injuries or chronic pain conditions, would enable a more thorough examination of individual and contextual factors. Additionally, a limitation related to the administration of the IPAQ should be considered. The IPAQ reflects physical activity performed during the previous seven days. However, it does not necessarily capture the level of activity at the time the experimental task was performed. Future studies should therefore take this aspect into account. Furthermore, the IPAQ is a self-reporting questionnaire, which introduces the possibility that the reported data may be inaccurate, a common limitation of questionnaire-based measures. In addition, the sample size of 44 participants, while sufficient for detecting main effects, remains relatively small for testing interaction effects with adequate statistical power. This should be kept in mind when interpreting null findings, including the non-significant effects of grip strength and standard PAL categories. Finally, we controlled entropy values of drawings with respect to stroke length but neglecting other factors that could impact drawings entropy such as movement velocity in performing the drawing. More kinematic variables could therefore be explored in further analyses.

## Conclusion

Our findings suggest that Local Shannon Entropy estimation of drawing strokes shows preliminary sensitivity of body representation and may therefore represent a promising exploratory tool for investigating the structural organization of the body schema. With respect to demographic and lifestyle variables, entropy of self-drawings tended to decrease with aging, suggesting that body representations may become less variable and more structured over time, potentially reflecting a progressively stabilized body schema. PA did not show a direct main effect when using standard IPAQ categories. However, when activity levels were examined using data-driven tertile classification, a significant interaction between age and physical activity emerged: this suggested that greater engagement in physical activity may be associated with maintaining more flexible or variable body representations despite aging. MET scores and grip strength did not show a direct association with body entropy values. These null findings should be interpreted as the relatively small sample size may have limited statistical power to detect such effects. One possible explanation is that the drawing task used in this study was relatively generic and not explicitly related to physical activity contexts. Future studies could therefore employ more context-specific drawing instructions (e.g., drawing oneself during or after training) to better capture potential links between physical capability and body schema representation. Overall, findings of this work highlight the potential of entropy-based analyses of digital drawings as a novel quantitative approach to studying body schema organization. Future research should further investigate additional predictors including cognitive factors, sensorimotor integration, and neuroplasticity, and extend this approach to developmental and clinical populations, such as individuals with neurological conditions or musculoskeletal pain.

## Data Availability

The datasets generated during and analyzed in the current study are available from the corresponding author upon reasonable request.
